# Reaching for the “first 95”: a cross-country analysis of HIV self-testing in 177,572 people in nine countries in sub-Saharan Africa

**DOI:** 10.1097/QAD.0000000000003106

**Published:** 2022-02-01

**Authors:** Eva Van Empel, Rebecca A. De Vlieg, Guy Harling, Maja E. Marcus, Kathleen Kahn, Till W. BÄRnighausen, Livia Montana, Augustine T. Choko, Jennifer Manne-Goehler

**Affiliations:** 1 Maastricht University, Maastricht, Netherlands; 2 Harvard Center for Population and Development Studies, Harvard University, Cambridge, United States; 3 Institute for Global Health, University College London, London, United Kingdom; 4 Department of Epidemiology, Harvard T.H. Chan School of Public Health, Boston, United States; 5 Africa Health Research Institute, KwaZulu-Natal, South Africa; 6 MRC/Wits Rural Public Health and Health Transitions Research Unit (Agincourt), University of the Witwatersrand, Johannesburg, South Africa; 7 University of Goettingen, Department of Economics and Centre for Modern Indian Studies, Goettingen, Germany; 8 INDEPTH Network, East Legon, Ghana; 9 Heidelberg University, Heidelberg Institute of Global Health (HIGH), Heidelberg, Germany; 10 Harvard T.H. Chan School of Public Health, Department of Global Health and Population, Boston, United States; 11 The Demographic and Health Surveys Program; 12 Malawi-Liverpool Wellcome Trust Clinical Research Programme, Blantyre, Malawi; 13 Massachusetts General Hospital, Division of Infectious Diseases, Boston, United States

**Keywords:** HIV, Self-testing, HIV testing, HIV seroprevalence, Sub-Saharan Africa

## Abstract

**Objectives:**

HIV self-testing (HIVST) offers a promising approach to increase HIV diagnosis and advance progress towards the UNAIDS 95-95-95 targets. We aimed to understand patterns of HIVST awareness and utilization in nine sub-Saharan African (SSA) countries, with the goal of identifying populations to target in disseminating this technology.

**Design:**

Cross-sectional study.

**Methods:**

We pooled individual-level population-based data from nine Demographic and Health Surveys (DHS) in SSA conducted 2015-2019 (Burundi, Cameroon, Guinea, Malawi, Senegal, Sierra Leone, South Africa, Zambia, Zimbabwe). Primary outcomes were HIVST awareness and utilization. We used logistic regression with survey fixed effects to explore the relationship between sociodemographic characteristics and these outcomes. Models were adjusted for sex, age, rural/urban residence, education, wealth, and marital status. We accounted for complex survey design.

**Results:**

The study sample included 177,572 people (66.0% women, mean age 29±10 years), of whom 86.6% (95%CI 86.4-86.7) were unaware of HIVST, 11.7% (95%CI 11.6-11.9) were aware of but never used HIVST, and 1.7% (95%CI 1.6-1.8) had used HIVST. In adjusted models, women were less likely to be aware of HIVST (OR 0.75, 95%CI 0.71-0.79), but more likely to have used HIVST (OR 1.17, 95%CI 1.03-1.32) compared to men. Rural residents, those who were least educated, and poorest were less likely to have heard of or used HIVST.

**Conclusions:**

HIVST awareness and uptake were low. Rural, less educated and lower income populations were least likely to have heard of or used HIVST. Efforts to scale-up HIVST in these settings should aim to reach these less advantaged groups.

## Introduction

HIV prevention programs have sought to reduce new HIV infections worldwide by promoting widespread HIV testing, linkage to care and ultimately high rates of viral suppression to prevent onward transmission. Recently, UNAIDS showed that only 76% of people with HIV in eastern and southern Africa – the global region with the highest HIV prevalence – knew their serostatus as of the end of 2017 [1]. HIV self-testing (HIVST) offers a promising approach to increase progress toward the 95-95-95 targets, which seek to ensure that 95% of people living with HIV (PLHIV) are aware of their serostatus, 95% of PLHIV receive antiretroviral therapy (ART) and 95% of those on ART are virally suppressed, by 2030 [2]. HIV self-tests have the advantage of providing a greater level of flexibility and privacy in contexts where HIV-related stigma is highly prevalent [3,4]. As such, HIVST offers an innovative approach to increase testing uptake among people who are reluctant to test in formal health care settings [5].

Given the potential benefits of HIVST, the World Health Organization (WHO) recommended as of 2016 that HIVST be offered as an additional HIV testing modality in this region [6]. Since the WHO published these guidelines, the Self-Test Africa (STAR) initiative has sought to increase HIVST in SSA and shape national policies that will promote more widespread scale-up of HIVST [7,8]. This initiative started in 2015 with implementation in three SSA countries (Malawi, Zambia, and Zimbabwe), followed by many others, and has resulted in 77 countries introducing policies that promote HIVST as of 2019 [8,9]. However, one study of HIVST awareness and uptake in Zimbabwe and Malawi found low levels of awareness (12.6%) and use (1.2%)[10], despite a high willingness to test (84.5%) among Zimbabwean men, the only sub-group in whom willingness was assessed [10]. Aside from this study, relatively little is known about the current levels of HIVST awareness and uptake in much of the region. In addition, to the best of our knowledge, there is little available evidence about the relationship between HIV-related stigma and HIVST, whereas prior research has shown that HIV-related stigma may be associated with reduced uptake of regular HIV testing [4,11].

In this study, we sought to evaluate awareness and utilization of HIVST among people 15 years or older in nine countries in SSA with variable HIV prevalence. Our secondary aim was to understand the factors that are correlated with the awareness and utilization of self-testing, including sociodemographic characteristics, and HIV-related stigma. The findings of this study could lead to potential targets for future intervention strategies to scale-up HIVST.

## Methods

### Data source

This study used data from nine Demographic and Health Surveys (DHS) conducted in SSA countries. The DHS Program provides technical assistance to countries for standardized household surveys which include the following population-based research topics: maternal and child health, nutrition, mortality, health services, malaria, and HIV [12]. DHS aims to provide high quality data for national and international planning and decision making [12]. We included surveys based on the following criteria: 1) the country was located within the SSA region; 2) the survey included questions about HIVST; and 3) HIV biomarker data were available. We included the most recent survey in each country. This led to a sample of nine surveys, from which we pooled individual-level data: Burundi (2016/2017), Cameroon (2018), Guinea (2018), Malawi (2015/2016), Senegal (2017), Sierra Leone (2019), South Africa (2016), Zambia (2018), and Zimbabwe (2015).

### Measures

The primary outcome measures were HIVST awareness and use. The questions were asked in the following forms: “Have you heard of test kits people can use to test themselves for HIV?” and “Have you ever tested yourself for HIV using a self-test kit?”. A secondary outcome was ever being tested for HIV: “I don't want to know the results, but have you ever been tested for HIV?”. Sociodemographic variables included sex (male/female), age (5-year age categories), type of residence (rural/urban), educational level (no education/primary/secondary/higher), wealth (poorest/poorer/middle/richer/richest), marital status (never in union/married/living with partner/widowed/divorced or separated), HIV status (negative/positive), and HIV-related stigma score (1-6). An HIV-related stigma score was created out of six separate questions about HIV-related stigma (Supplemental Digital Content (SDC) 1), as has been done previously in studies using the DHS to interrogate HIV-related stigma [13].

### Statistical analyses

Women aged 15-49 and men aged 15-54 were included, as these were the age groups that were available in all countries. Analyses were limited to the participants who responded to the HIVST questions, except for “ever tested for HIV”, where the total study population was included in the analyses, as all participants responded to this question. Second, proportions of HIVST awareness and utilization were explored by participant characteristics such as sex, age, rural/urban residence, educational level, wealth, marital status, HIV status, and HIV-related stigma. Third, correlates of HIV self-testing behavior were explored in two multivariable logistic regression analyses with survey fixed effects. The first model (“Model 1”) was adjusted for age, sex, educational level, household wealth and marital status. A second model (“Model 2”) also included HIV-related stigma. Fourth, we additionally performed modified Poisson regression analysis and present prevalence ratios for Models 1 and 2.

We conducted three supplementary analyses. First, we assessed variation in awareness and use of self-testing at the country level by performing disaggregated regression analyses by country. Second, in order to compare HIVST use with regular HIV testing, we re-ran our multivariable regression model for the outcome of having ever tested for HIV. Third, we explored whether outcomes of HIVST use are related to the level of HIVST awareness, therefore we conducted multivariable regressions for HIVST use, but only among those who were also aware of HIVST. Analyses were performed in SPSS and STATA. A complex sample package was used to account for the complex survey design. Standard DHS survey weights were used to adjust for non-response and sample imbalance. In this study we present unweighted numbers and weighted percentages.

## Results

### Baseline characteristics

The total study sample consisted of 192,712 respondents, of which 177,572 people (92.6%) responded to the HIVST questions. Sociodemographic differences between responders and non-responders can be found in a Supplementary Appendix (see Table, SDC 2). Among those who responded to the HIVST questions, 66.0% (n=117,127) were women, the mean age was 29 ± 10 years (Table 1) and HIV prevalence in this population was 6.2% (n=7,033) (Table 2). Of this pooled sample, 63.9% (95% CI 63.6-64.1) had ever been tested for HIV, 13.4% (95% CI 13.3-13.6) were aware of HIVST and only 1.7% (95% CI 1.6-1.8) had ever used a self-test kit to test for HIV (Table 1). Of the people who were aware of HIVST, a pooled estimate of 12.7% had ever used HIVST (Table 1).

### Awareness of HIVST

**Figure 1:**
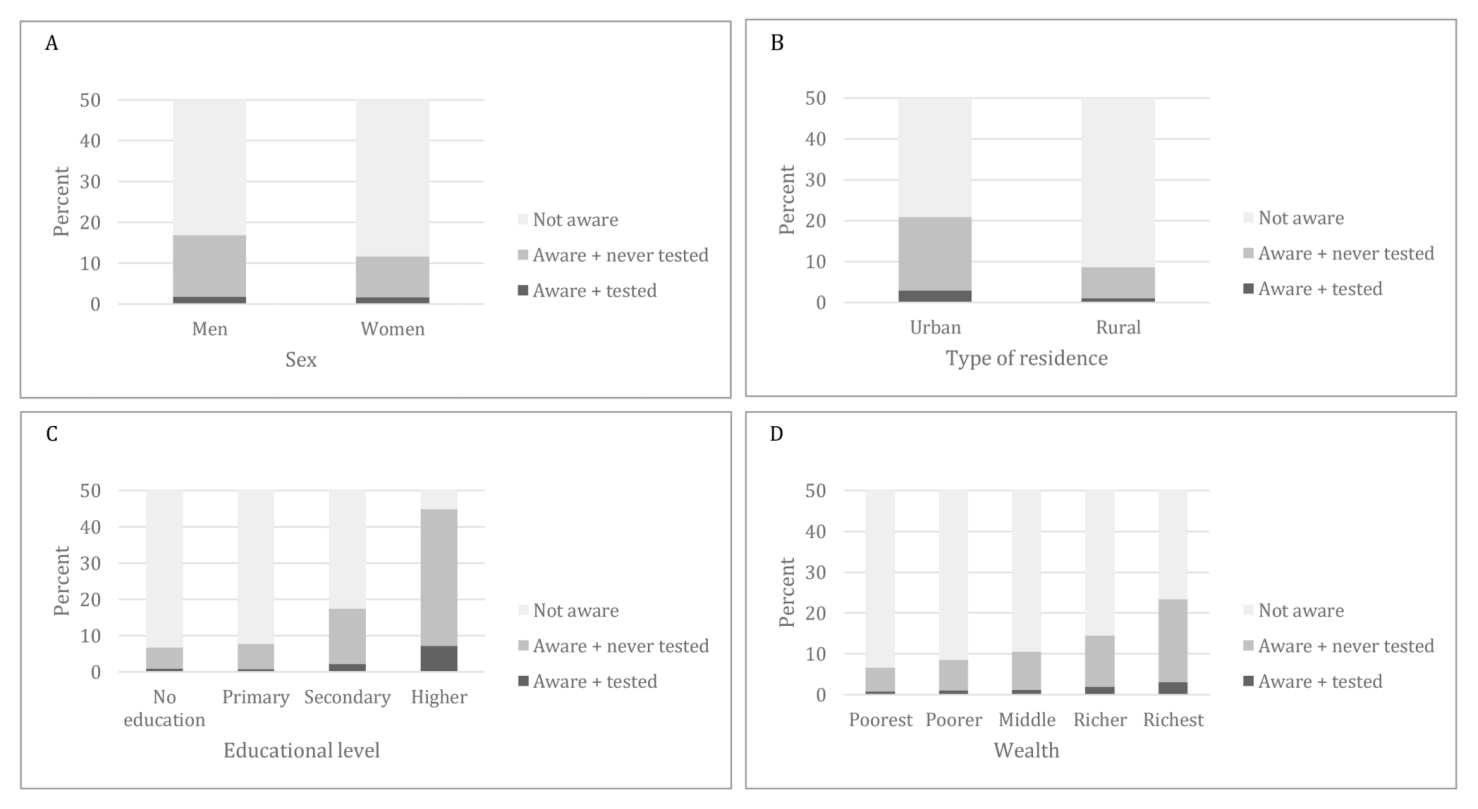
Proportions of HIV self-testing awareness and utilization per A) sex, B) type of residence, C) educational level, and D) wealth index

Proportions of HIVST awareness by sociodemographic characteristics can be found in a Supplementary Appendix (see Table, SDC 3). In multivariate regression models we found that women (OR 0.75, 95% CI 0.71-0.79), young adolescents (15-19 years: OR 1.00 vs. 50-54 years: OR 1.67, 95% CI 1.45-1.94), and people living in rural areas (OR 0.81, 95% CI 0.75-0.88) were less likely to be aware of HIVST than men, older age groups, and urban residents, respectively (Table 3, SDC 4). Moreover, there were significant differences in the association between HIVST awareness and educational level (no education vs. primary: OR 1.03, 95% CI 0.96-1.11; secondary: 1.81, 95% CI 1.68-1.95; higher: OR 4.89, 95% CI 4.45-5.37) and wealth (poorest vs. poorer: OR 1.26, 95% CI 1.16-1.37; middle: OR 1.45, 95% CI 1.32-1.58; richer: OR 1.70, 95% CI 1.54-1.88; richest: OR 2.36, 95% CI 2.12-2.62) with less educated and less wealthy people being less aware of HIVST (Fig 1, Table 3). When adding HIV-related stigma to the model (Model 2, n=166,089), stigma was significantly inversely associated with HIVST awareness (0 vs. 6: OR 0.82, 95% CI 0.70-0.94) (Table 3). Prevalence ratios showed similar results to odds ratios (Table 3, SDC 4).

### Use of HIVST

We display the proportions using HIVST use overall and by key sociodemographic characteristics in a Supplementary Appendix (see Table, SDC 3). Multivariate logistic regression analysis showed women had greater odds of having ever used HIVST compared to men (OR 1.17, 95% CI 1.03-1.32) (Table 4). Moreover, we found that young adolescents (15-19 years: OR 1.00 vs. 50-54 years: OR 1.86, 95% CI 1.23-2.80), rural residents (OR 0.74, 95% CI 0.62-0.89), those with lower educational attainment (no education vs. primary: OR 0.79, 95% CI 0.65-0.97; secondary: OR 1.64, 95% CI 1.36-1.98; higher: OR 4.20, 95% CI 3.43-5.16), and less wealthy people (poorest vs. poorer: OR 1.28, 95% CI 1.04-1.59; middle: OR 1.22, 95% CI 0.96-1.55; richer: OR 1.48, 95% CI 1.17-1.86, richest: OR 1.66, 95% CI 1.31-2.11) were less likely to have used HIVST compared to older age groups, urban residents, higher educated, and wealthier people, respectively (Fig 1, Table 4, SDC 4). The second model additionally included HIV-related stigma (Model 2, n=166,089) and showed that, consistent with HIVST awareness, people who self-reported a high level of HIV-related stigma were less likely to have ever used a self-test (0: OR 1.00 vs. 6: OR 0.23, 95% CI 0.15-0.35) (Table 4). Prevalence ratios showed similar results to odds ratios (Table 4, SDC 4).

### Country-level differences and supplementary analyses

Regression analyses of HIVST awareness and use disaggregated by country showed results were largely stable across countries, with few notable exceptions. First, men in Sierra Leone and urban residents in Senegal were less likely to be aware of HIVST compared to women and rural residents, respectively. For HIVST use we found that women had lower odds of having ever used HIVST in Cameroon. Moreover, we found HIVST use was greater in wealthier people in many countries, whereas we found the opposite relationship in Sierra Leone (see Figure, SDC 5; see Tables, SDC 6). Country fixed effects showed that Cameroon, Sierra Leone, South Africa and Zambia are leading countries with respect to both awareness and use of HIVST (see Table, SDC 6). Multivariable regression models investigating “ever tested for HIV” and sociodemographic characteristics showed similar results to the findings for HIVST use, further details are described in a Supplementary Appendix (see Tables, SDC 7). We additionally investigated HIVST use among those who are aware. Overall, regression analyses showed similar patterns in terms of HIVST use when restricting to those who were aware of HIVST, as for HIVST use among the entire study population (see Table, SDC 8).

## Discussion

This study of pooled individual-level data across nine nationally representative population-based surveys in SSA demonstrated that less than one in seven people were aware of HIVST and far fewer had ever used HIVST. We found that less advantaged populations, including those that are rural, less educated and lower income, were less likely to be aware of or use HIVST, further reinforcing inequality in access to important new testing modalities that can improve timely linkage to needed HIV care. These findings not only highlight an important, untapped opportunity to speed progress toward the “first 95;” that is, the UNAIDS target that 95% of people know their HIV status, but also offer specific policy-relevant insight about how to target dissemination of this technology [2].

These findings are important because HIV diagnosis is a necessary precursor to treatment and viral suppression, which can in turn prevent disease transmission [14]. As reported in recent studies, HIVST improves HIV testing uptake in general [9,15]. Our results showed that implementation of HIVST is still far from achieving its maximal potential, with 98% of the study population having never self-tested. The consistent increase in self-testing across wealth and educational levels suggest that focusing on traditionally disadvantaged groups has the potential to increase HIVST uptake overall. This is especially important given that these lower socioeconomic groups have been shown to have a higher risk of acquiring HIV [16]. Additionally, these interventions should aim to reach rural populations. Moreover, we found that results of HIVST use were comparable to those for usual modalities of HIV testing, indicating these two testing methods might be reaching similar populations. Our findings are consistent with Johnson *et al*[10] but show that they are generalizable across nine countries in SSA – countries in this study represent about 40.7% of the HIV epidemic in the SSA region (see Table, SDC 9) [17,18]. Our finding that these less advantaged groups are also less likely to use HIVST are also similar to a recent single-country study undertaken in rural Malawi [19]. Literature about HIVST use and awareness outside of SSA has shown low HIVST awareness (14%) in Northern Thailand, though nearly 40% of MSM in Beijing, China had used HIVST in one study [20,21].

Our study demonstrates a gap between HIVST knowledge and uptake. It is important to understand how this gap has emerged, in order to improve HIVST implementation. As such, future research should focus on identifying what factors prevent people who are aware of HIVST from self-testing. Greater awareness of these barriers could inform the design of programs and policies that can translate HIVST awareness into actual use. Prior studies report that barriers to HIVST include HIVST costs, concerns about parents finding out they are sexually active, the fear of a positive test result and perceived unreliability of the test [22,23]. These concerns may contribute to the low self-testing rates found in this study.

In addition, we examined HIV-related stigma because HIVST, in particular because of privacy considerations of testing at home, might be particularly attractive for people who have a more stigmatized view of HIV. Interestingly, we did not observe higher self-testing rates among this group, indeed we found the opposite relationship. This finding could have multiple explanations. First, people with high levels of HIV-related stigma might not self-test because they avoid any type of HIV-related testing due to shame or resentment around this subject [11,13]. Alternatively, people with high levels of stigma might not admit to self-testing, as they do not want to be associated with the disease.

Since the WHO recommended self-testing as an additional HIV testing service in 2016[6], countries in SSA have begun to develop national policies to implement and disseminate this technology. Thus, it is important to acknowledge that these surveys were conducted during a period when most countries had policies that were recently introduced or still in development [24–28]. In a Supplementary Appendix we provided a brief overview of HIVST access per country at the time these surveys were conducted (see Table, SDC 10). This lack of access may be one reason for the low rates of HIVST awareness and use in this population. However, our study showed that a meaningful proportion of people did have access to self-test kits, perhaps in part through distribution of HIVST via validation trials or internet-based ordering [29,30].

This study has several important strengths and limitations. First, an important strength of this study is the large sample size. Second, the survey questions used in this study are evaluated broadly and have high response rates. The latter limits the risk of non-response bias; because DHS covers wide-ranging topics, people might not necessarily decline because of HIV-related arguments. However, while DHS questions are consistent across surveys, they have not been validated as a true measure of HIVST awareness or utilization in these populations. Another limitation of this study is that we used self-reported outcomes that may be subject to both response bias and recall bias; people might not answer truthfully or may not remember past events accurately. Furthermore, data were limited to certain SSA countries, as not all SSA countries had recent DHS available and the two HIVST questions were not asked in all surveys. Thus, while the findings are robust across these nine countries, it is not clear to what extent they will be generalizable to all countries in this region.

In conclusion, HIVST awareness in this population is limited and a very small proportion of people have ever used HIVST. Across all contexts, less advantaged groups such as rural, poor and less educated populations have also been neglected in the dissemination of HIVST. Future interventions should seek to expand HIVST services in SSA with a particular focus on these least advantaged groups and with the goal to advance progress toward achieving the “first 95.” Finally, a greater understanding of what drives the observed knowledge-uptake gap for HIVST will be critical to maximize the potential of this promising new testing modality.

## Supplementary Material

Supplementary Material

## Figures and Tables

**Table 1:** Survey characteristics^1^

Country	Year	Sample size	% Female	Mean age ± SD	% HIV positive	% Ever tested (95% CI)	% Aware of HIVST (95% CI)	% Use of HIVST (95% CI)	HIVST use/awareness proportion^2^
**Burundi**	2016/2017	23 553	70.0%	29 ± 10	0.9%	64.6 (64.0-65.2)	4.1 (3.8-4.3)	0.3 (0.2-0.4)	7.3%
**Cameroon**	2018	19 422	67.6%	28 ± 10	2.7%	68.3 (67.7-69.0)	16.5 (16.0-17.1)	2.6 (2.4-2.9)	15.8%
**Guinea**	2018	12 200	71.4%	29 ±10	1.6%	19.3 (18.7-20.0)	8.7 (8.3-9.3)	0.8 (0.7-1.0)	9.2%
**Malawi**	2015/2016	31 481	76.4%	28 ± 10	9.0%	81.8 (81.3-82.2)	10.6 (10.2-10.9)	1.0 (0.9-1.1)	9.4%
**Senegal**	2017	22 199	71.3%	28 ± 10	0.5%	43.0 (42.4-43.7)	5.3 (5.0-5.6)	0.2 (0.1-0.2)	3.8%
**Sierra Leone**	2019	20 923	69.1%	29 ± 10	1.8%	49.0 (48.3-49.7)	20.9 (20.4-21.5)	3.5 (3.3-3.8)	16.7%
**South Africa**	2016	11 481	71.0%	30 ± 10	22.2%	83.4 (82.7-84.1)	25.3 (24.5-26.1)	3.0 (2.7-3.4)	11.9%
**Zambia**	2018	24 986	53.5%	29 ± 10	11.4%	84.0 (83.6-84.5)	20.7 (20.2-21.2)	2.9 (2.7-3.1)	14.0%
**Zimbabwe**	2015	11 327	26.8%	28 ± 11	11.1%	58.4 (57.5-59.3)	14.8 (14.2-15.5)	1.4 (1.2-1.7)	9.5%
**Total**		177 572	66.0%	29 ± 10	6.2%	63.9 (63.6-64.1)	13.4 (13.3-13.6)	1.7(1.6-1.8)	12.7%

* Abbreviations: SD= of HIVST x 100%. standard deviation; HIVST= HIV self-testing.

1 Percentages are weighted with DHS sampling weights, numbers are presented unweighted.

2 HIVST proportion= use of HIVST / awareness

**Table 2:** Participant characteristics of the pooled sample^1^

	**N**	**% of population**
**Sex**		
Men	60 445	34.0%
Women	117 127	66.0%
**Age groups**		
15-19 years	40 410	22.3%
20-24 years	31 998	18.0%
25-29 years	28 153	16.2%
30-34 years	24 096	13.8%
35-39 years	20 741	11.8%
40-44 years	15 796	8.9%
45-49 years	12 944	7.2%
50-54 years^2^	3 434	1.9%
**Residence type**		
Urban	68 254	39.6%
Rural	109 318	60.4%
**Highest educational level** ^3^		
No education	41 352	23.2%
Primary	57 159	32.3%
Secondary	69 277	38.5%
Higher	9 782	6.0%
**Household wealth index**		
Poorest	31 094	16.9%
Poorer	33 444	18.2%
Middle	36 220	19.4%
Richer	36 741	21.4%
Richest	40 073	24.1%
**Marital status**		
Never in union	65 155	36.4%
Married	90 628	51.3%
Living with partner	9 637	5.5%
Widowed	3 251	1.8%
Divorced/separated	8 901	5.1%
**HIV status** ^4^		
HIV-	106 108	93.8%
HIV+	7 033	6.2%
**HIV-related stigma score** ^5,6^		
0	9 417	5.5%
1	20 894	12.4%
2	36 112	21.6%
3	42 870	26.5%
4	23 569	14.3%
5	16 585	9.9%
6	16 644	9.9%
**Total**	177 572	100.0%

1 Percentages are weighted with DHS sampling weights, numbers are presented unweighted.

2 The age group 50-54 years only includes male participants.

3 Total number of responses= 178 541 (100.0%); missing responses= 1 (0.0%).

4 Total number of participants who consented to HIV testing= 113 271 (63.4%); not consented to HIV testing= 65 270 (36.6%).

5 Total number of responses= 167 082 (93.6%); not asked in the South African survey (n= 11 459, 6.4%).

6 The HIV-related stigma score consists of six questions, one point was given for every question answered with ‘yes’, indicating the presence of HIV-related stigma.

**Table 3:** Multivariable logistic regression analysis of the association between awareness of HIVST and participant characteristics from DHS surveys across nine countries in SSA^1,2^

	Awareness of HIVST			
	Model 1		Model 2	
	OR (95% CI)	PR (95% CI)	OR (95% CI)	PR(95% CI)
**Sex**				
Men	REF	REF	REF	REF
Women	0.75 (0.71-0.79)	0.80 (0.78-0.82)	0.74 (0.70-0.79)	0.79 (0.77-0.81)
**Residence type**				
Urban	REF	REF	REF	REF
Rural	0.81 (0.75-0.88)	0.89 (0.86-0.93)	0.83 (0.76-0.91)	0.91 (0.87-0.94)
**Highest educational level**				
No education	REF	REF	REF	REF
Primary	1.03 (0.96-1.11)	1.04 (0.99-1.09)	1.03 (0.96-1.11)	1.04 (0.98-1.09)
Secondary	1.81 (1.68-1.95)	1.69 (1.61-1.77)	1.78 (1.65-1.92)	1.65 (1.57-1.74)
Higher	4.89 (4.45-5.37)	3.13 (2.96-3.31)	4.84 (4.39-5.35)	3.07 (2.89-3.26)
**Household wealth index**				
Poorest	REF	REF	REF	REF
Poorer	1.26 (1.16-1.37)	1.22 (1.15-1.29)	1.23 (1.12-1.34)	1.20 (1.13-1.27)
Middle	1.45 (1.32-1.58)	1.38 (1.30-1.45)	1.40 (1.27-1.54)	1.34 (1.26-1.42)
Richer	1.70 (1.54-1.88)	1.57 (1.48-1.66)	1.62 (1.45-1.80)	1.51 (1.42-1.60)
Richest	2.36 (2.12-2.62)	2.01 (1.89-2.13)	2.29 (2.04-2.57)	1.97 (1.85-2.10)
**HIV stigma severity score**				
0			REF	REF
1			0.96 (0.87-1.06)	0.97 (0.91-1.04)
2			1.00 (0.91-1.09)	0.98 (0.94-1.06)
3			1.06 (0.97-1.16)	1.05 (0.99-1.12)
4			1.01 (0.91-1.12)	0.99 (0.93-1.06)
5			0.84 (0.74-0.95)	0.84 (0.78-0.91)
6			0.82 (0.70-0.94)	0.82 (0.76-0.88)
**Total number of respondents**	177 570		166 089	

Abbreviations: HIVST= HIV self-testing; OR= Odds ratio; CI= Confidence Interval; PR= Prevalence ratio.

1 Analyses were performed using DHS sample weights, total number of respondents are presented unweighted.

2 Analyses were additionally adjusted for age and marital status.

**Table 4:** Multivariable logistic regression analysis of the association between use of HIVST and participant characteristics from DHS surveys across nine countries in SSA^1,2^

	**Use of HIVST**			
	**Model 1**		**Model 2**	
	**OR (95% CI)**	**PR (95% CI)**	**OR** (**95% CI)**	**PR (95% CI)**
**Sex**				
Men	REF	REF	REF	REF
Women	1.17 (1.03-1.32)	1.18 (1.09-1.29)	1.21 (1.07-1.38)	1.18 (1.08-1.29)
**Residence type**				
Urban	REF	REF	REF	REF
Rural	0.74 (0.62-0.89)	0.78 (0.70-0.87)	0.76 (0.62-0.92)	0.78 (0.70-0.88)
**Highest educational level**				
No education	REF	REF	REF	REF
Primary	0.79 (0.65-0.97)	0.83 (0.71-0.96)	0.78 (0.63-0.96)	0.79 (0.68-0.93)
Secondary	1.64 (1.36-1.98)	1.64 (1.44-1.88)	1.56 (1.29-1.90)	1.54 (1.34-1.77)
Higher	4.20 (3.43-5.16)	4.12 (3.53-4.81)	3.72 (3.01-4.60)	3.57 (3.04-4.19)
**Household wealth index**				
Poorest	REF	REF	REF	REF
Poorer	1.28 (1.04-1.59)	1.16 (0.99-1.37)	1.16 (0.92-1.46)	1.07 (0.90-1.27)
Middle	1.22 (0.96-1.55)	1.17 (1.00-1.38)	1.09 (0.85-1.41)	1.05 (0.88-1.24)
Richer	1.48 (1.17-1.86)	1.38 (1.17-1.62)	1.33 (1.04-1.69)	1.24 (1.04-1.49)
Richest	1.66 (1.31-2.11)	1.61 (1.35-1.91)	1.51 (1.18-1.95)	1.47 (1.22-1.78)
**HIV stigma severity score**				
0			REF	REF
1			0.98 (0.75-1.29)	0.92 (0.76-1.12)
2			1.06 (0.82-1.38)	0.94 (0.78-1.13)
3			1.15 (0.90-1.47)	1.02 (0.86-1.22)
4			1.48 (1.12-1.96)	1.43 (1.18-1.72)
5			0.44 (0.31-0.63)	0.44 (0.35-0.56)
6			0.23 (0.15-0.35)	0.20 (0.15-0.27)
**Total number of respondents**	177 570		166 089	

Abbreviations: HIVST= HIV self-test’s; OR= Odds ratio; CI = Confidence Interval; PR= Prevalence ratio.

1 Analyses were performed using DHS sample weights, total number of respondents are presented unweighted.

2 Analyses were additionally adjusted for age and marital status.

## Data Availability

DHS data is publicly available by request at the DHS website: https://dhsprogram.com/data/available-datasets.cfm.
