# Supplementary material for: Reaching for the “first 95”: a cross-country analysis of HIV self-testing in 177,572 people in nine countries in sub-Saharan Africa
Source: AIDS. Author manuscript; Available in PMC 2022 Feb 1. (PMC7612158; doi:10.1097/QAD.0000000000003106)
Supplement: Supplementary Material [file EMS136194-supplement-Supplementary_Material.docx]

**Supplemental Digital Content**

***Supplemental Digital Content 1 – Questions about HIV-related stigma (Text)***

An HIV-related stigma score was created out of six separate questions about HIV-related stigma. Participants were asked to answer with yes or no to the following six statements: “Would be ashamed if someone in the family had HIV”, “would buy vegetables from vendor with HIV”, “children with HIV should be allowed to attend school with children without HIV”, “people hesitate to take HIV test because reaction of other people if positive”, “people talk badly about people with or believed to have HIV”, and “people with or believed to have HIV lose respect from other people”. All variables were recoded into binary variables, where “yes” indicated the presence of HIV-related stigma. The sum of affirmative responses was used to form a 6-item stigma scale. The questions about HIV-related stigma were asked in all surveys except South Africa.

***Supplemental Digital Content 2 – Differences between responders and non-responders (Table)***

|  | **Responders** | | **Non-responders** | | **Chi-square** |
| --- | --- | --- | --- | --- | --- |
|  | **N** | **%** | **N** | **%** | ***p*-value** |
| **Sex** |  |  |  |  | <0**.**001** |
| Men | 60 445 | 34**.**0% | 1 522 | 9.7% |  |
| Women | 117 127 | 66**.**0% | 13 618 | 90.3% |  |
| **Age** **groups** |  |  |  |  | <0**.**001** |
| 15-19 years | 40 410 | 22**.**3% | 3 886 | 25.8% |  |
| 20-24 years | 31 998 | 18**.**0% | 2 356 | 15.3% |  |
| 25-29 years | 28 153 | 16**.**2% | 2 247 | 14.7% |  |
| 30-34 years | 24 096 | 13**.**8% | 2 127 | 14.1% |  |
| 35-39 years | 20 741 | 11**.**8% | 1 773 | 12.0% |  |
| 40-44 years | 15 796 | 8**.**9% | 1 489 | 9.8% |  |
| 45-49 years | 12 944 | 7**.**2% | 1 181 | 7.6% |  |
| 50-54 years^2^ | 3 434 | 1**.**9% | 81 | 0.5% |  |
| **Country** |  |  |  |  | <0**.**001** |
| Burundi | 23 553 | 13.2% | 896 | 6.3% |  |
| Cameroon | 19 422 | 11.0% | 540 | 3.6% |  |
| Guinea | 12 200 | 6.9% | 2 525 | 16.7% |  |
| Malawi | 31 481 | 17.6% | 559 | 4.3% |  |
| Senegal | 22 199 | 12.7% | 1 269 | 5.8% |  |
| Sierra Leone | 20 923 | 11.8% | 1 461 | 8.8% |  |
| South Africa | 11 481 | 6.4% | 437 | 3.3% |  |
| Zambia | 24 986 | 14.0% | 429 | 2.8% |  |
| Zimbabwe | 11 327 | 6.4% | 7 024 | 48.5% |  |
| **Residence type** |  |  |  |  | <0**.**001** |
| Urban | 68 254 | 39**.**6% | 4 837 | 29.0% |  |
| Rural | 109 318 | 60**.**4% | 10 303 | 71.0% |  |
| **Highest educational level** |  |  |  |  | <0**.**001** |
| No education | 41 352 | 23**.**2% | 4 857 | 30.2% |  |
| Primary | 57 159 | 32**.**3% | 3 793 | 26.1% |  |
| Secondary | 69 277 | 38**.**5% | 5 744 | 39.0% |  |
| Higher | 9 782 | 6**.**0% | 746 | 4.7% |  |
| **Household** **wealth** **index** |  |  |  |  | <0**.**001** |
| Poorest | 31 094 | 16**.**9% | 3 803 | 25.2% |  |
| Poorer | 33 444 | 18**.**2% | 3 141 | 21.5% |  |
| Middle | 36 220 | 19**.**4% | 2 720 | 18.9% |  |
| Richer | 36 741 | 21**.**4% | 2 728 | 17.3% |  |
| Richest | 40 073 | 24**.**1% | 2 748 | 17.1% |  |
| **Marital status** |  |  |  |  | <0**.**001** |
| Never in union | 65 155 | 36**.**4% | 4 606 | 29.9% |  |
| Married | 90 628 | 51**.**3% | 8 610 | 56.7% |  |
| Living with partner | 9 637 | 5**.**5% | 505 | 3.5% |  |
| Widowed | 3 251 | 1**.**8% | 488 | 3.4% |  |
| Divorced/separated | 8 901 | 5**.**1% | 931 | 6.4% |  |
| **HIV** **status** |  |  |  |  | <0**.**001** |
| HIV- | 106 108 | 93**.**8% | 9 427 | 87.3% |  |
| HIV+ | 7 033 | 6**.**2% | 1 379 | 12.7% |  |
| **HIV stigma severity score** |  |  |  |  | <0**.**001** |
| 0 | 9 417 | 5**.**5% | 775 | 11.7% |  |
| 1 | 20 894 | 12**.**4% | 1 633 | 24.1% |  |
| 2 | 36 112 | 21**.**6% | 1 794 | 25.9% |  |
| 3 | 42 870 | 26**.**5% | 1 982 | 28.1% |  |
| 4 | 23 569 | 14**.**3% | 538 | 7.8% |  |
| 5 | 16 585 | 9**.**9% | 130 | 1.9% |  |
| 6 | 16 644 | 9**.**9% | 28 | 0.4% |  |
| **Total number of respondents** | 177 572 | 100**.**0% |  |  |  |

Abbreviations: HIVST= HIV self-testing.

**p* < **.**05. ** *p* < **.**001. ^1^Percentages are weighted with DHS sampling weights, numbers are presented unweighted. ^2^The age group 50-54 years only includes male participants.

***Supplemental Digital Content 3 – Proportions of HIVST (Table)***

**Table 2**

*Proportions of participant characteristics, and HIV-related stigma among people who are not aware of HIVST, are aware but have never tested with HIVST and people who have tested with HIVST^1^*

|  | **HIVST** | | | | | | | |  | |  |
| --- | --- | --- | --- | --- | --- | --- | --- | --- | --- | --- | --- |
|  | **Not aware** | | **Aware + not tested** | | | **Aware + tested** | | | **Chi-square** | |  |
|  | **N** | **%** | | **N** | **%** | | **N** | **%** | | ***p*-value** | |
| **Sex** |  |  | |  |  | |  |  | | <0**.**001** | |
| Men | 50 545 | 83**.**2% | | 8 920 | 15**.**1% | | 980 | 1**.**8% | |  | |
| Women | 103 770 | 88**.**3% | | 11 493 | 10**.**0% | | 1 864 | 1**.**7% | |  | |
| **Age** **groups** |  |  | |  |  | |  |  | | <0**.**001** | |
| 15-19 years | 37 086 | 91**.**6% | | 3 047 | 7**.**6% | | 277 | 0**.**8% | |  | |
| 20-24 years | 27 668 | 86**.**1% | | 3 809 | 12**.**1% | | 521 | 1**.**8% | |  | |
| 25-29 years | 23 732 | 84**.**0% | | 3 814 | 13**.**8% | | 607 | 2**.**2% | |  | |
| 30-34 years | 20 437 | 84**.**4% | | 3 147 | 13**.**4% | | 512 | 2**.**2% | |  | |
| 35-39 years | 17 715 | 85**.**0% | | 2 626 | 13**.**0% | | 400 | 2**.**0% | |  | |
| 40-44 years | 13 577 | 85**.**9% | | 1 930 | 12**.**3% | | 289 | 1**.**8% | |  | |
| 45-49 years | 11 225 | 86**.**3% | | 1 530 | 12**.**2% | | 189 | 1**.**5% | |  | |
| 50-54 years^2^ | 2 875 | 84**.**1% | | 510 | 14**.**4% | | 49 | 1**.**5% | |  | |
| **Country** |  |  | |  |  | |  |  | | <0**.**001** | |
| Burundi | 22 600 | 95**.**9% | | 878 | 3**.**8% | | 75 | 0**.**3% | |  | |
| Cameroon | 16 241 | 83**.**5% | | 2 680 | 13**.**9% | | 501 | 2**.**6% | |  | |
| Guinea | 11 203 | 91**.**3% | | 896 | 7**.**9% | | 101 | 0**.**8% | |  | |
| Malawi | 28 123 | 89**.**4% | | 3 055 | 9**.**6% | | 303 | 1**.**0% | |  | |
| Senegal | 20 916 | 94**.**7% | | 1 232 | 5**.**1% | | 51 | 0**.**2% | |  | |
| Sierra Leone | 16 421 | 79**.**1% | | 3 759 | 17**.**4% | | 743 | 3**.**5% | |  | |
| South Africa | 8 840 | 74**.**7% | | 2 317 | 22**.**2% | | 324 | 3**.**0% | |  | |
| Zambia | 20 362 | 79**.**3% | | 4 051 | 17**.**8% | | 573 | 2**.**9% | |  | |
| Zimbabwe | 9 609 | 85**.**2% | | 1 545 | 13**.**4% | | 173 | 1**.**4% | |  | |
| **Residence type** |  |  | |  |  | |  |  | | <0**.**001** | |
| Urban | 54 978 | 79**.**1% | | 11 518 | 18**.**1% | | 1 758 | 2**.**8% | |  | |
| Rural | 99 337 | 91**.**5% | | 8 895 | 7**.**6% | | 1 086 | 0**.**9% | |  | |
| **Highest educational level** |  |  | |  |  | |  |  | | <0**.**001** | |
| No education | 38 438 | 93**.**3% | | 2 526 | 5**.**8% | | 388 | 0**.**9% | |  | |
| Primary | 52 760 | 92**.**3% | | 3 995 | 7**.**0% | | 404 | 0**.**7% | |  | |
| Secondary | 57 719 | 82**.**6% | | 10 218 | 15**.**3% | | 1 340 | 2**.**1% | |  | |
| Higher | 5 396 | 55**.**2% | | 3 674 | 37**.**7% | | 712 | 7**.**1% | |  | |
| **Household** **wealth** **index** |  |  | |  |  | |  |  | | <0**.**001** | |
| Poorest | 28 969 | 93**.**4% | | 1 868 | 5**.**8% | | 257 | 0**.**8% | |  | |
| Poorer | 30 483 | 91**.**5% | | 2 616 | 7**.**5% | | 345 | 1**.**1% | |  | |
| Middle | 32 290 | 89**.**5% | | 3 502 | 9**.**3% | | 428 | 1**.**2% | |  | |
| Richer | 31 465 | 85**.**5% | | 4 612 | 12**.**6% | | 664 | 1**.**9% | |  | |
| Richest | 31 108 | 76**.**6% | | 7 815 | 20**.**3% | | 1 150 | 3**.**0% | |  | |
| **Marital status** |  |  | |  |  | |  |  | | <0**.**001** | |
| Never in union | 56 404 | 86**.**1% | | 7 774 | 12**.**2% | | 977 | 1**.**7% | |  | |
| Married | 79 054 | 86**.**9% | | 10 084 | 11**.**4% | | 1 490 | 1**.**7% | |  | |
| Living with partner | 8 327 | 86**.**4% | | 1 123 | 11**.**6% | | 187 | 1**.**9% | |  | |
| Widowed | 2 899 | 89**.**3% | | 325 | 9**.**9% | | 27 | 0**.**9% | |  | |
| Divorced/separated | 7 631 | 85**.**3% | | 1 107 | 12**.**8% | | 163 | 1**.**9% | |  | |
| **HIV** **status** |  |  | |  |  | |  |  | | <0**.**001** | |
| HIV- | 92 211 | 86**.**6% | | 12 233 | 11**.**7% | | 1 664 | 1**.**7% | |  | |
| HIV+ | 5 709 | 79**.**7% | | 1 167 | 17**.**9% | | 157 | 2**.**4% | |  | |
| **HIV stigma severity score** |  |  | |  |  | |  |  | | <0**.**001** | |
| 0 | 8 130 | 86**.**1% | | 1 134 | 12**.**3% | | 153 | 1**.**6% | |  | |
| 1 | 18 209 | 86**.**8% | | 2 383 | 11**.**7% | | 302 | 1**.**6% | |  | |
| 2 | 32 217 | 88**.**9% | | 3 477 | 9**.**8% | | 418 | 1**.**3% | |  | |
| 3 | 36 686 | 85**.**4% | | 5 412 | 12**.**6% | | 772 | 2**.**0% | |  | |
| 4 | 20 494 | 86**.**9% | | 2 422 | 10**.**4% | | 653 | 2**.**7% | |  | |
| 5 | 14 853 | 89**.**6% | | 1 585 | 9**.**6% | | 147 | 0**.**9% | |  | |
| 6 | 14 886 | 89**.**3% | | 1 683 | 10**.**2% | | 75 | 0**.**5% | |  | |
| **Total number of respondents** | 154 315 |  | | 20 413 |  | | 2 844 |  | |  | |

Abbreviations: HIVST= HIV self-testing.

**p* < **.**05. ** *p* < **.**001. ^1^Percentages are weighted with DHS sampling weights, numbers are presented unweighted. ^2^The age group 50-54 years only includes male participants.

**Awareness of HIVST**

Proportions of participant characteristics, HIV-related stigma, and HIVST awareness are presented in Supplemental Digital Content 3, Table 2. HIVST awareness was lower for women (11.7%, compared to 16.9% men, *p* < 0.001), young adolescents (15-19 years= 8.4% vs. 50-54 year= 15.9%, *p* < 0.001), rural residents (8.5% vs. 20.1% urban residents, *p* < 0.001), people who were less educated (no education= 6.7% vs. higher= 44.8%, *p* < 0.001), people in the poorest wealth quintile (poorest= 6.6% vs. richest= 23.3%, *p* < 0.001), and people who did not have HIV (13.4% vs. 20.3% of those living with HIV, *p* < 0.001). Finally, people were also less likely to be aware of HIVST if they scored higher on the HIV-related stigma scale (0= 13.9% vs. 6= 10.7%, *p* < 0.001).

**Use of HIVST**

Proportions of HIVST utilization by participant characteristics, and HIV-related stigma can be found in Supplemental Digital Content 3, Table 2. Among those who ever used a self-test, sex appeared to be significantly different, but differences were minor (1.7% women vs. 1.8% men, *p* < 0.001). Those who were less likely to have ever tested themselves for HIV using a self-test kit were young adolescents (15-19 years= 0.8% vs. 50-54 years= 1.5%; *p* < 0.001), rural residents (0.9% vs. 2.8% urban residents, *p* < 0.001), people who were less educated (no education= 0.9% vs. higher=7.1%, *p* < 0.001), people who were less wealthy (poorest= 0.8 vs. richest= 3.0%, *p* < 0.001), and people who were not infected with HIV (1.7% vs. 2.4% of people with HIV, *p* < 0.001) (Supplemental Digital Content 3, Table 2; Fig 1). Last, there was no clear trend in self-testing rates and the HIV-related stigma score, but people with high HIV-related stigma reported lower levels of HVIST use (0= 1.6%; vs. 6= 0.5%, *p* < 0.001) (Supplemental Digital Content 3, Table 2).

***Supplemental Digital Content 4 – Multivariable logistic regression, full version (Tables)***

**Table 3**

*Multivariable logistic regression analysis of the association between awareness of HIVST and participant characteristics from DHS surveys across nine countries in SSA^1^*

|  | **Awareness of HIVST** | | | |
| --- | --- | --- | --- | --- |
|  | **Model 1** | | **Model 2** | |
|  | **OR** **(95% CI)** | **PR (95% CI)** | **OR** (**95% CI)** | **PR (95% CI)** |
| **Sex** |  |  |  |  |
| Men | REF | REF | REF | REF |
| Women | 0**.**75 (0**.**71-0**.**79) | 0.80 (0.78-0.82) | 0**.**74 (0**.**70-0**.**79) | 0.79 (0.77-0.81) |
| **Age** **groups** |  |  |  |  |
| 15-19 years | REF | REF | REF | REF |
| 20-24 years | 1**.**56 (1**.**46-1**.**68) | 1.50 (1.44-1.58) | 1.53 (1.42-1.64) | 1.49 (1.42-1.56) |
| 25-29 years | 1**.**82 (1**.**69-1**.**97) | 1.72 (1.63-1.81) | 1.82 (1.67-1.98) | 1.72 (1.63-1.82) |
| 30-34 years | 1**.**90 (1**.**75-2**.**07) | 1.78 (1.68-1.87) | 1.91 (1.75-2.10) | 1.78 (1.68-1.89) |
| 35-39 years | 1**.**88 (1**.**72-2**.**05) | 1.76 (1.66-1.87) | 1.88 (1.71-2.07) | 1.78 (1.67-1.89) |
| 40-44 years | 1**.**80 (1**.**64-1**.**98) | 1.74 (1.63-1.85) | 1.83 (1.65-2.03) | 1.75 (1.64-1.88) |
| 45-49 years | 1**.**75 (1**.**59-1**.**94) | 1.66 (1.55-1.78) | 1.78 (1.60-1.99) | 1.70 (1.58-1.83) |
| 50-54 years^2^ | 1**.**67 (1**.**45-1**.**94) | 1.68 (1.52-1.85) | 1.72 (1.47-2.00) | 1.71 (1.54-1.90) |
| **Residence type** |  |  |  |  |
| Urban | REF | REF | REF | REF |
| Rural | 0**.**81 (0**.**75-0**.**88) | 0.89 (0.86-0.93) | 0**.**83 (0**.**76-0**.**91) | 0.91 (0.87-0.94) |
| **Highest educational level** |  |  |  |  |
| No education | REF | REF | REF | REF |
| Primary | 1**.**03 (0**.**96-1**.**11) | 1.04 (0.99-1.09) | 1**.**03 (0**.**96-1**.**11) | 1.04 (0.98-1.09) |
| Secondary | 1**.**81 (1**.**68-1**.**95) | 1.69 (1.61-1.77) | 1**.**78 (1**.**65-1**.**92) | 1.65 (1.57-1.74) |
| Higher | 4**.**89 (4**.**45-5**.**37) | 3.13 (2.96-3.31) | 4**.**84 (4**.**39-5**.**35) | 3.07 (2.89-3.26) |
| **Household** **wealth** **index** |  |  |  |  |
| Poorest | REF | REF | REF | REF |
| Poorer | 1**.**26 (1**.**16-1**.**37) | 1.22 (1.15-1.29) | 1**.**23 (1**.**12-1**.**34) | 1.20 (1.13-1.27) |
| Middle | 1**.**45 (1**.**32-1**.**58) | 1.38 (1.30-1.45) | 1**.**40 (1**.**27-1**.**54) | 1.34 (1.26-1.42) |
| Richer | 1**.**70 (1**.**54-1**.**88) | 1.57 (1.48-1.66) | 1**.**62 (1**.**45-1**.**80) | 1.51 (1.42-1.60) |
| Richest | 2**.**36 (2**.**12-2**.**62) | 2.01 (1.89-2.13) | 2**.**29 (2**.**04-2**.**57) | 1.97 (1.85-2.10) |
| **Marital status** |  |  |  |  |
| Never in union | REF | REF | REF | REF |
| Married | 1**.**06 (1**.**00-1**.**12) | 1.01 (0.97-1.04) | 1**.**05 (0**.**99-1**.**12) | 1.00 (0.96-1.04) |
| Living with partner | 1**.**06 (0**.**96-1**.**17) | 1.05 (0.99-1.12) | 1**.**02 (0**.**91-1**.**15) | 1.03 (0.96-1.11) |
| Widowed | 0**.**94 (0**.**80-1**.**10) | 0.94 (0.84-1.05) | 0**.**94 (0**.**79-1**.**11) | 0.94 (0.84-1.06) |
| Divorced/separated | 1**.**16 (1**.**06-1**.**28) | 1.08 (1.01-1.15) | 1**.**11 (1**.**01-1**.**23) | 1.04 (0.97-1.12) |
| **HIV stigma severity score** |  |  |  |  |
| 0 | .. | .. | REF | REF |
| 1 |  |  | 0**.**96 (0**.**87-1**.**06) | 0.97 (0.91-1.04) |
| 2 |  |  | 1**.**00 (0**.**91-1**.**09) | 0.98 (0.94-1.06) |
| 3 |  |  | 1**.**06 (0**.**97-1**.**16) | 1.05 (0.99-1.12) |
| 4 |  |  | 1.01 (0**.**91-1**.**12) | 0.99 (0.93-1.06) |
| 5 |  |  | 0**.**84 (0**.**74-0**.**95) | 0.84 (0.78-0.91) |
| 6 |  |  | 0**.**82 (0**.**70-0**.**94) | 0.82 (0.76-0.88) |
| **Total number of respondents** | 177 570 | | 166 089 | |

Abbreviations: HIVST= HIV self-testing; OR= Odds ratio; CI= Confidence Interval; PR= Prevalence ratio.

^1^Analyses were performed using DHS sample weights, total number of respondents are presented unweighted. ^2^The age group 50-54

years only includes male participants.

**Table 4**

*Multivariable logistic regression analysis of the association between use of HIVST and participant characteristics from DHS surveys across nine countries in SSA^1^*

|  | **Use of HIVST** | | | |
| --- | --- | --- | --- | --- |
|  | **Model 1** | | **Model 2** | |
|  | **OR** **(95% CI)** | **PR (95% CI)** | **OR** (**95% CI)** | **PR (95% CI)** |
| **Sex** |  |  |  |  |
| Men | REF | REF | REF | REF |
| Women | 1**.**17 (1.03-1.32) | 1.18 (1.09-1.29) | 1.21 (1.07-1.38) | 1.18 (1.08-1.29) |
| **Age** **groups** |  |  |  |  |
| 15-19 years | REF | REF | REF | REF |
| 20-24 years | 1.96 (1.61-2.38) | 2.01 (1.73-2.34) | 1.86 (1.51-2.27) | 1.94 (1.66-2.27) |
| 25-29 years | 2.21 (1.79-2.71) | 2.49 (2.13-2.91) | 2.16 (1.73-2.70) | 2.47 (2.09-2.92) |
| 30-34 years | 2.38 (1.90-2.97) | 2.68 (2.27-3.18) | 2.30 (1.80-2.94) | 2.60 (2.17-3.12) |
| 35-39 years | 2.28 (1.82-2.87) | 2.55 (2.14-3.05) | 2.21 (1.73-2.83) | 2.52 (2.08-3.05) |
| 40-44 years | 2.19 (1.72-2.79) | 2.56 (2.12-3.09) | 2.23 (1.72-2.89) | 2.57 (2.10-3.15) |
| 45-49 years | 1.89 (1.46-2.45) | 2.10 (1.70-2.58) | 1.82 (1.37-2.41) | 2.09 (1.67-2.62) |
| 50-54 years^2^ | 1.86 (1.23-2.80) | 1.99 (1.44-2.76) | 1.84 (1.19-2.84) | 2.00 (1.43-2.80) |
| **Residence type** |  |  |  |  |
| Urban | REF | REF | REF | REF |
| Rural | 0.74 (0.62-0.89) | 0.78 (0.70-0.87) | 0.76 (0.62-0.92) | 0.78 (0.70-0.88) |
| **Highest educational level** |  |  |  |  |
| No education | REF | REF | REF | REF |
| Primary | 0.79 (0.65-0.97) | 0.83 (0.71-0.96) | 0.78 (0.63-0.96) | 0.79 (0.68-0.93) |
| Secondary | 1.64 (1.36-1.98) | 1.64 (1.44-1.88) | 1.56 (1.29-1.90) | 1.54 (1.34-1.77) |
| Higher | 4.20 (3.43-5.16) | 4.12 (3.53-4.81) | 3.72 (3.01-4.60) | 3.57 (3.04-4.19) |
| **Household** **wealth** **index** |  |  |  |  |
| Poorest | REF | REF | REF | REF |
| Poorer | 1.28 (1.04-1.59) | 1.16 (0.99-1.37) | 1.16 (0.92-1.46) | 1.07 (0.90-1.27) |
| Middle | 1.22 (0.96-1.55) | 1.17 (1.00-1.38) | 1.09 (0.85-1.41) | 1.05 (0.88-1.24) |
| Richer | 1.48 (1.17-1.86) | 1.38 (1.17-1.62) | 1.33 (1.04-1.69) | 1.24 (1.04-1.49) |
| Richest | 1.66 (1.31-2.11) | 1.61 (1.35-1.91) | 1.51 (1.18-1.95) | 1.47 (1.22-1.78) |
| **Marital status** |  |  |  |  |
| Never in union | REF | REF | REF | REF |
| Married | 1.07 (0.93-1.23) | 1.06 (0.95-1.18) | 1.07 (0.92-1.25) | 1.05 (0.94-1.18) |
| Living with partner | 1.08 (0.86-1.37) | 1.11 (0.94-1.31) | 1.02 (0.80-1.31) | 1.08 (0.90-1.31) |
| Widowed | 0.57 (0.35-0.92) | 0.55 (0.37-0.82) | 0.50 (0.30-0.84) | 0.52 (0.34-0.80) |
| Divorced/separated | 1.07 (0.85-1.35) | 1.10 (0.92-1.31) | 1.00 (0.78-1.29) | 1.01 (0.84-1.23) |
| **HIV stigma severity score** |  |  |  |  |
| 0 | .. | .. | REF | REF |
| 1 |  |  | 0.98 (0.75-1.29) | 0.92 (0.76-1.12) |
| 2 |  |  | 1.06 (0.82-1.38) | 0.94 (0.78-1.13) |
| 3 |  |  | 1.15 (0.90-1.47) | 1.02 (0.86-1.22) |
| 4 |  |  | 1.48 (1.12-1.96) | 1.43 (1.18-1.72) |
| 5 |  |  | 0.44 (0.31-0.63) | 0.44 (0.35-0.56) |
| 6 |  |  | 0.23 (0.15-0.35) | 0.20 (0.15-0.27) |
| **Total number of respondents** | 177 570 | | 166 089 | |

Abbreviations: HIVST= HIV self-test’s; OR= Odds ratio; CI= Confidence Interval; PR= Prevalence ratio.

^1^Analyses were performed using DHS sample weights, total number of respondents are presented unweighted. ^2^The age group 50-54

years only includes male participants.

***Supplemental Digital Content 5 – HIV self-testing awareness and utilization by country (Figure)***

**Figure 1. Proportions of HIV self-testing awareness and utilization per country**

***Supplemental Digital Content 6 – HIV self-testing regression analyses disaggregated by country (Tables)***

**Table 5**

*Multivariable regression analysis by country to examine the association between awareness of HIVST and participant characteristics*

|  | **Awareness of HIVST** | | | | | | | | | | | | | | | | | |
| --- | --- | --- | --- | --- | --- | --- | --- | --- | --- | --- | --- | --- | --- | --- | --- | --- | --- | --- |
|  | **West-Africa** | | | | | | **Eastern and Southern Africa** | | | | | | | | | | | |
|  | **Guinea** | | **Senegal** | | **Sierra Leone** | | **Burundi** | | **Cameroon** | | **Malawi** | | **South Africa** | | **Zambia** | | **Zimbabwe** | |
|  | **OR** | **95% CI** | **OR** | **95% CI** | **OR** | **95% CI** | **OR** | **95% CI** | **OR** | **95% CI** | **OR** | **95% CI** | **OR** | **95% CI** | **OR** | **95% CI** | **OR** | **95% CI** |
| **Sex**  Men  Women | REF  1.16 | 0.89-1.51 | REF  1.25 | 1.00-1.56 | REF  1.22 | 1.07-1.41 | REF  0.51 | 0.41-0.63 | REF  0.54 | 0.45-0.64 | REF  0.71 | 0.63-0.81 | REF  0.80 | 0.69-.93 | REF  0.57 | 0.50-0.64 | REF  0.55 | 0.46-0.65 |
| **Age** **groups**  15-19  20-24  25-29  30-34  35-39  40-44  45-49  50-54^2^ | REF  1.53  2.13  2.12  2.04  2.11  2.44  1.13 | 1.17-2.00  1.57-2.89  1.56-2.90  1.46-2.84  1.40-3.18  1.61-3.69  0.57-2.23 | 1.15  1.15  1.35  1.52  1.28  1.42  2.12 | 0.91-1.44  0.88-1.51  1.03-1.76  1.13-2.06  0.94-1.75  1.02-1.98  1.20-3.74 | REF  1.58  2.14  2.33  1.97  1.98  2.02  1.86 | 1.34-1.85  1.85-2.47  1.93-2.83  1.61-2.41  1.58-2.49  1.62-2.52  1.28-2.69 | REF  1.65  1.60  1.76  1.74  1.61  1.70  1.60 | 1.29-2.12  1.16-2.21  1.28-2.43  1.21-2.50  1.08-2.40  0.97-2.98  0.91-2.80 | REF  1.80  2.51  2.53  2.70  2.83  2.83  3.44 | 1.47-2.20  2.02-3.11  1.97-3.24  2.07-3.51  2.16-3.71  2.08-3.84  2.36-5.02 | REF  1.34  1.51  1.57  1.52  1.37  1.20  1.30 | 1.09-1.65  1.17-1.95  1.23-2.01  1.14-2.01  1.03-1.82  0.88-1.64  0.75-2.26 | REF  1.91  1.80  1.71  1.75  1.551.49  1.20 | 1.53-2.37  1.41-2.28  1.34-2.18  1.38-2.23  1.19-2.01  1.13-1.98  0.75-1.93 | REF  1.62  1.73  1.91  1.84  1.95  1.94  1.71 | 1.37-1.92  1.40-2.13  1.51-2.41  1.48-2.28  1.53-2.50  1.52-2.47  1.29-2.27 | REF  1.54  2.52  2.52  2.70  2.03  1.81  1.84 | 1.23-1.95  1.90-3.35  1.92-3.31  1.99-3.67  1.41-2.92  1.24-2.62  1.19-2.83 |
| **Residence type**  Urban  Rural | REF  0.76 | 0.55-1.05 | REF  1.64 | 1.28-2.10 | REF  1.10 | 0.85-1.43 | REF  0.58 | 0.42-0.79 | REF  0.94 | 0.73-1.21 | REF  0.71 | 0.60-0.85 | REF  0.74 | 0.63-0.88 | REF  0.79 | 0.64-0.98 | REF  0.72 | 0.57-0.92 |
| **Highest educational level**  No education  Primary  Secondary  Higher | REF  1.72  3.48  9.21 | 1.29-2.31  2.67-4.53  6.79-12.50 | REF  1.32  1.85  4.80 | 1.10-1.58  1.52-2.26  3.50-6.59 | REF  1.03  1.68  4.72 | 0.87-1.21  1.45-1.94  3.88-5.74 | REF  0.94  1.52  2.93 | 0.76-1.17  1.14-2.02  1.83-4.69 | REF  2.62  4.80  11.57 | 1.91-3.59  3.55-6.48  8.33-16.09 | REF  1.03  1.65  5.44 | 0.84-1.27  1.34-2.03  3.78-7.82 | REF  1.12  2.41  5.61 | 0.60-2.08  1.40-4.15  3.18-9.88 | REF  1.55  2.53  7.39 | 1.17-2.04  1.91-3.35  5.47-9.99 | REF  1.44  2.47  7.49 | 0.54-3.87  0.93-6.56  2.81-19.95 |
| **Household** **wealth** **index**  Poorest  Poorer  Middle  Richer  Richest | REF  0.78  1.21  1.09  1.45 | 0.53-1.15  0.82-1.80  0.72-1.66  0.93-2.26 | REF  1.38  1.72  1.77  1.33 | 1.05-1.83  1.25-2.36  1.23-2.55  0.89-2.00 | REF  1.04  1.24  1.30  1.79 | 0.85-1.28  0.97-1.57  0.98-1.74  1.26-2.54 | REF  1.00  0.99  1.05  1.78 | 0.73-1.35  0.71-1.37  0.75-1.48  1.26-2.53 | REF  1.55  2.07  2.67  3.79 | 1.06-2.26  1.39-3.08  1.75-4.08  2.48-5.77 | REF  1.11  1.13  1.09  1.72 | 0.93-1.32  0.94-1.35  0.88-1.34  1.40-2.11 | REF  1.46  1.68  2.24  2.69 | 1.18-1.81  1.34-2.12  1.74-2.88  2.02-3.58 | REF  1.60  2.03  3.41  5.19 | 1.33-1.94  1.67-2.46  2.69-4.33  4.06-6.63 | REF  1.59  1.47  1.87  2.72 | 1.20-2.11  1.09-1.98  1.33-2.61  1.89-3.92 |
| **Marital status**  Never in union  Married  Living with partner  Widowed  Divorced/  separated | REF  1.28  1.94  1.19  1.34 | 1.02-1.62  1.18-3.18  0.58-2.44  0.81-2.23 | REF  1.43  0.15  1.25  1.50 | 1.15-1.77  0.02-1.34  0.44-3.57  0.90-2.49 | REF  0.88  0.85  0.73  0.96 | 0.77-1.01  0.66-1.09  0.50-1.06  0.74-1.24 | REF  0.75  0.99  0.78  1.22 | 0.57-0.97  0.74-1.32  0.38-1.63  0.76-1.97 | REF  1.00  0.83  0.88  1.06 | 0.85-1.17  0.67-1.02  0.56-1.40  0.86-1.31 | REF  1.19  1.23  1.05  1.18 | 0.99-1.44  0.91-1.65  0.68-1.61  0.92-1.51 | REF  1.10  1.23  0.88  1.69 | 0.94-1.30  0.99-1.51  0.53-1.47  1.25-2.30 | REF  1.02  1.24  1.05  1.19 | 0.89-1.16  0.65-2.36  0.74-1.47  0.95-1.49 | REF  0.95  1.15  1.16  1.00 | 0.76-1.18  0.66-2.00  0.62-2.15  0.72-1.38 |
| **Total (weighted)** |  | 12 340 |  | 22 635 |  | 21 130 |  | 23 532 |  | 19 587 |  | 31 424 |  | 11 459 |  | 25 046 |  | 11 388 |
| **Total (unweighted)** |  | 12 200 |  | 22 199 |  | 20 923 |  | 23 553 |  | 19 422 |  | 31 481 |  | 11 481 |  | 24 986 |  | 11 327 |

^1^Analyses were performed using DHS sample weights. ^2^The age group 50-54 years only includes male participants.

**Table 6**

*Multivariable regression analysis by country to examine the association between use of HIVST and participant characteristics*

|  | **Use of HIVST** | | | | | | | | | | | | | | | | | |
| --- | --- | --- | --- | --- | --- | --- | --- | --- | --- | --- | --- | --- | --- | --- | --- | --- | --- | --- |
|  | **West-Africa** | | | | | | **Eastern and Southern Africa** | | | | | | | | | | | |
|  | **Guinea** | | **Senegal** | | **Sierra Leone** | | **Burundi** | | **Cameroon** | | **Malawi** | | **South Africa** | | **Zambia** | | **Zimbabwe** | |
|  | **OR** | **95% CI** | **OR** | **95% CI** | **OR** | **95% CI** | **OR** | **95% CI** | **OR** | **95% CI** | **OR** | **95% CI** | **OR** | **95% CI** | **OR** | **95% CI** | **OR** | **95% CI** |
| **Sex**  Men  Women | REF  2.97 | 1.56-5.67 | REF  2.62 | 0.53-12.81 | REF  2.34 | 1.72-3.18 | REF  0.79 | 0.44-1.43 | REF  0.72 | 0.54-0.95 | REF  0.96 | 0.63-1.46 | REF  0.98 | 0.65-1.49 | REF  1.06 | 0.87-1.29 | REF  0.78 | 0.48-1.26 |
| **Age** **groups**  15-19  20-24  25-29  30-34  35-39  40-44  45-49  50-54^2^ | REF  2.50  3.53  3.80  4.11  2.56  3.22  2.56 | 1.29-4.84  1.94-6.42  1.90-7.60  1.83-9.26  0.86-7.61  0.98-10.54  0.30-21.64 | REF  0.91  4.36  2.55  2.49  2.92  0.35  37.74 | 0.15-5.58  0.72-26.58  0.51-12.77  0.34-18.39  0.39-21.93  0.02-5.41  4.33-329.05 | REF  2.20  2.21  3.15  2.23  2.96  1.66  1.49 | 1.54-3.14  1.52-3.21  1.97-5.03  1.44-3.47  1.79-4.92  0.99-2.79  0.57-3.93 | REF  4.79  8.03  8.57  6.62  3.24  10.31  1.34 | 1.55-14.81  2.08-30.94  2.00-36.73  1.09-40.13  0.57-18.35  1.87-56.67  0.11-15.71 | REF  2.09  3.44  2.96  3.42  3.70  3.64  3.53 | 1.26-3.49  2.08-5.67  1.72-5.08  1.95-5.99  2.11-6.49  2.10-6.30  1.59-7.83 | REF  1.22  1.27  1.57  2.00  1.05  0.42  2.04 | 0.69-2.16  0.65-2.48  0.76-3.24  0.98-4.08  0.48-2.33  0.12-1.46  0.46-8.94 | REF  2.56  2.24  2.43  2.31  1.63  1.87  0.84 | 1.31-5.01  1.21-4.15  1.33-4.44  1.16-4.59  0.77-3.46  0.93-3.74  0.15-4.57 | REF  1.84  1.84  1.55  1.41  1.60  1.60  0.90 | 1.22-2.77  1.12-3.03  0.92-2.62  0.83-2.39  0.95-2.69  0.88-2.92  0.35-2.30 | REF  1.59  1.89  2.99  3.15  2.24  2.26  2.34 | 0.75-3.36  0.96-3.74  1.51-5.93  1.48-6.69  1.03-4.88  0.93-5.50  0.85-6.49 |
| **Residence type**  Urban  Rural | REF  0.39 | 0.10-1.47 | REF  1.12 | 0.35-3.60 | REF  0.73 | 0.46-1.16 | REF  0.66 | 0.25-1.77 | REF  0.85 | 0.57-1.28 | REF  0.52 | 0.37-0.72 | REF  0.81 | 0.56-1.17 | REF  0.70 | 0.47-1.02 | REF  1.51 | 0.85-2.69 |
| **Highest educational level**  No education  Primary  Secondary  Higher | REF  1.57  6.09  7.37 | 0.73-3.34  3.14-11.80  3.86-14.07 | REF  1.00  4.19  8.49 | 0.29-3.41  1.82-9.64  3.08-23.42 | REF  0.93  1.44  6.92 | 0.66-1.33  1.04-1.99  5.12-9.35 | REF  0.99  5.77  8.11 | 0.39-2.52  2.55-13.04  2.06-31.95 | REF  1.54  3.34  7.39 | 0.89-2.67  19.4-5.75  4.13-13.21 | REF  0.84  1.79  4.85 | 0.44-1.62  0.91-3.49  2.28-10.32 | REF  0.17  0.55  1.65 | 0.05-0.61  0.19-1.60  0.56-4.89 | REF  1.00  1.50  2.94 | 0.50-1.98  0.74-3.05  1.41-6.12 | REF  3.50  5.24  10.76 | 0.45-27.26  0.69-39.77  1.34-86.44 |
| **Household** **wealth** **index**  Poorest  Poorer  Middle  Richer  Richest | REF  2.95  5.13  4.16  3.24 | 0.50-17.46  1.08-24.26  0.64-27.14  0.49-21.40 | REF  2.00  2.12  1.70  3.35 | 0.46-8.62  0.52-8.62  0.28-10.34  0.70-16.08 | REF  1.00  0.91  0.65  0.36 | 0.70-1.42  0.58-1.44  0.41-1.05  0.21-0.60 | REF  1.65  0.89  2.00  2.57 | 0.31-8.85  0.15-5.19  0.42-9.54  0.55-12.03 | REF  0.62  0.88  1.42  1.95 | 0.32-1.20  0.45-1.72  0.74-2.72  1.00-3.81 | REF  1.92  1.17  1.42  1.93 | 1.00-3.68  0.59-2.34  0.76-2.68  1.04-3.58 | REF  2.18  2.02  2.27  2.27 | 1.15-4.12  1.04-3.91  1.11-4.63  1.06-4.86 | REF  1.38  1.83  3.39  4.41 | 0.81-2.34  1.05-3.20  1.85-6.21  2.38-8.18 | REF  2.59  1.40  2.63  4.95 | 1.13-5.95  0.54-3.62  1.07-6.46  1.75-14.02 |
| **Marital status**  Never in union  Married  Living with partner  Widowed  Divorced/  separated | REF  1.58  1.67  1.92  2.49 | 0.79-3.16  0.55-5.02  0.24-15.70  0.66-9.40 | REF  1.33  0.00  3.75  3.74 | 0.50-3.56  0.00-0.00  0.35-39.91  0.65-21.45 | REF  0.86  1.89  0.28  0.90 | 0.65-1.15  1.25-2.86  0.11-0.68  0.53-1.52 | REF  1.02  0.40  0.50  0.44 | 0.46-2.29  0.12-1.37  0.05-4.77  0.05-4.05 | REF  1.24  0.79  0.77  1.16 | 0.93-1.66  0.54-1.15  0.29-2.02  0.72-1.85 | REF  1.28  1.36  0.67  1.71 | 0.73-2.25  0.60-3.11  0.13-3.33  0.85-3.45 | REF  1.25  1.22  1.30  1.46 | 0.85-1.86  0.68-2.20  0.39-4.33  0.75-2.83 | REF  1.04  1.75  0.52  0.93 | 0.75-1.44  0.46-6.64  0.17-1.62  0.57-1.51 | REF  1.04  0.23  0.14  1.27 | 0.65-1.66  0.03-1.74  0.02-1.11  0.60-2.69 |
| **Total (weighted)** |  | 12 340 |  | 22 635 |  | 21 130 |  | 23 532 |  | 19 587 |  | 31 424 |  | 11 459 |  | 25 046 |  | 11 388 |
| **Total (unweighted)** |  | 12 200 |  | 22 199 |  | 20 923 |  | 23 553 |  | 19 422 |  | 31 481 |  | 11 481 |  | 24 986 |  | 11 327 |

^1^Analyses were performed using DHS sample weights. ^2^The age group 50-54 years only includes male participants.

**Table 7**

*Country effect estimates^1,2^*

|  | **Aware of HIVST** | **Use of HIVST** | **Ever tested for HIV** |
| --- | --- | --- | --- |
|  | **OR** **(95% CI)** | **OR** (**95% CI)** | **OR** (**95% CI)** |
| Burundi  Cameroon  Guinea  Malawi  Senegal  Sierra Leone  South Africa  Zambia  Zimbabwe | REF  3.22 (2.77-3.75)  1.83 (1.56-2.15)  2.64 (2.31-3.03)  1.02 (0.86-1.22)  5.07 (4.33-5.94)  4.95 (4.26-5.75)  4.49 (3.88-5.20)  2.42 (2.09-2.79) | REF  5.49 (3.82-7.88)  2.01 (1.26-3.19)  2.90 (2.03-4.16)  0.42 (0.25-0.73)  8.33 (5.66-12.25)  5.18 (3.54-7.59)  6.69 (4.54-9.86)  3.12 (2.09-4.65) | REF  1.00 (0.91-1.08)  0.07 (0.06-0.08)  2.31 (2.16-2.46)  0.31 (0.28-0.33)  0.37 (0.34-0.40)  2.25 (2.00-2.52)  3.66 (3.35-4.00)  1.42 (1.30-1.56) |
| **Total number of respondents** | 177 570 | 177 570 | 192 710 |

Abbreviations: HIVST= HIV self-test’s; OR= Odds ratio; CI= Confidence Interval.

^1^Analyses were performed using DHS sample weights, total number of respondents are presented unweighted. ^2^Additionally adjusted for sex, age, residence type, educational level, wealth, and marital status.

***Supplemental Digital Content 7 – Ever tested for HIV (Tables)***

**Table 8**

*Proportions of participant characteristics, and HIV-related stigma between people who had ever tested for HIV compared to those who have never tested^1^*

|  | **Ever tested for HIV** | | | | |
| --- | --- | --- | --- | --- | --- |
|  | **No** | | **Yes** | | **Chi-square** |
|  | **N** | **%** | N | **%** | ***p*-value** |
| **Sex**  Men  Women | 29 268  43 376 | 47.1%  32.8% | 32 699  87 369 | 52.9%  67.2% | <0.001** |
| **Age** **groups**  15-19 years  20-24 years  25-29 years  30-34 years  35-39 years  40-44 years  45-49 years  50-54 years^2^ | 29 755  11 287  7 535  6 053  5 931  5 137  5 383  1 563 | 67.6%  32.7%  24.7%  22.6%  26.0%  29.1%  38.0%  44.3% | 14 541  23 067  22 865  20 170  16 583  12 148  8 742  1 952 | 32.4%  67.3%  75.3%  77.4%  74.0%  70.9%  62.0%  55.7% | <0.001** |
| **Country**  Burundi  Cameroon  Guinea  Malawi  Senegal  Sierra Leone  South Africa  Zambia  Zimbabwe | 9 340  6 515  12 424  6 152  14 044  12 378  2 380  4 632  4 779 | 37.8%  33.4%  83.8%  19.8%  58.5%  53.8%  20.0%  17.3%  26.7% | 15 109  13 447  2 301  25 888  9 424  10 006  9 538  20 783  13 572 | 62.2%  66.6%  16.2%  80.2%  41.5%  46.2%  80.0%  82.7%  73.3% | <0.001** |
| **Residence type**  Urban  Rural | 24 685  47 959 | 33.8%  39.6% | 48 406  71 662 | 66.2%  60.4% | <0.001** |
| **Highest educational level**  No education  Primary  Secondary  Higher | 25702  20 159  25 030  1 752 | 54.0%  33.2%  33.6%  17.8% | 20 507  40 793  49 991  8 776 | 46.0%  66.8%  66.4%  82.2% | <0.001** |
| **Household** **wealth** **index**  Poorest  Poorer  Middle  Richer  Richest | 15 783  15 090  14 978  13 781  13 012 | 43.9%  40.5%  38.1%  34.8%  31.8% | 19 114  21 495  23 962  25 688  29 809 | 56.1%  59.5%  61.9%  65.2%  68.2% | <0.001** |
| **Marital status**  Never in union  Married  Living with partner  Widowed  Divorced/separated | 39 454  28 687  1 820  888  1 795 | 57.3%  27.9%  18.4%  23.3%  18.2% | 30 307  70 551  8 322  2 851  8 037 | 42.7%  72.1%  81.6%  76.7%  81.8% | <0.001** |
| **HIV** **status**  HIV-  HIV+ | 46 600  922 | 40.1%  10.7% | 68 935  7 490 | 59.9%  89.3% | <0.001** |
| **HIV stigma severity score**  0  1  2  3  4  5  6 | 2 589  5 640  10 640  13 083  10 428  9 324  10 737 | 26.0%  25.2%  28.4%  29.3%  42.7%  55.2%  63.7% | 7 603  16 887  27 266  31 769  13 679  7 391  5 935 | 74.0%  74.8%  71.6%  70.7%  57.3%  44.8%  36.3% | <0.001** |
| **Total number of respondents**^3^ | 72 644 |  | 120 068 |  |  |

**p* < .05. ** *p* < .001. ^1^Percentages are weighted with DHS sampling weights, numbers are presented unweighted. ^2^The age group 50-54 years only includes male participants. ^3^The total number of respondents (n=192 712) differs from the total study sample (n=172 572), as more people answered the “ever tested for HIV” question than the HIVST question.

**Table 9**

*Multivariable logistic regression analysis with country fixed effects to examine the association between ever tested for HIV and participant characteristics from pooled data of nine DHS surveys^1^*

|  | **Ever tested for HIV** | |
| --- | --- | --- |
|  | **Model 1** | **Model 2** |
|  | **OR** **(95% CI)** | **OR** (**95% CI)** |
| **Sex**  Men  Women | REF  2.60 (2.51-2.70) | REF  2.88 (2.77-2.99) |
| **Age** **groups**  15-19  20-24  25-29  30-34  35-39  40-44  45-49  50-54^2^ | REF  3.65 (3.48-3.82)  5.04 (4.76-5.33)  5.07 (4.77-5.39)  4.21 (3.95-4.49)  3.37 (3.15-3.60)  2.20 (2.05-2.37)  2.36 (2.10-2.64) | REF  3.59 (3.41-3.77)  4.79 (4.50-5.09)  4.76 (4.46-5.08)  3.79 (3.54-4.06)  2.99 (2.79-3.21)  1.93 (1.78-2.08)  2.03 (1.80-2.28) |
| **Residence type**  Urban  Rural | REF  0.82 (0.77-0.87) | REF  0.82 (0.77-0.88) |
| **Highest educational level**  No education  Primary  Secondary  Higher | REF  1.67 (1.58-1.75)  2.90 (2.75-3.06)  5.17 (4.67-5.74) | REF  1.54 (1.46-1.62)  2.53 (2.39-2.68)  4.61 (4.14-5.15) |
| **Household** **wealth** **index**  Poorest  Poorer  Middle  Richer  Richest | REF  1.26 (1.20-1.33)  1.48 (1.40-1.57)  1.61 (1.51-1.71)  1.63 (1.52-1.76) | REF  1.24 (1.18-1.32)  1.44 (1.35-1.52)  1.51 (1.41-1.61)  1.57 (1.45-1.70) |
| **Marital status**  Never in union  Married  Living with partner  Widowed  Divorced/separated | REF  4.34 (4.12-4.57)  4.99 (4.60-5.42)  4.05 (3.61-4.55)  3.86 (3.56-4.19) | REF  4.95 (4.69-5.23)  5.86 (5.37-6.38)  4.59 (4.06-5.18)  4.19 (3.85-4.56) |
| **HIV-related stigma score**  0  1  2  3  4  5  6 | .. | REF  1.20 (1.11-1.30)  1.26 (1.16-1.36)  1.28 (1.19-1.39)  1.12 (1.03-1.22)  0.90 (0.82-0.98)  0.74 (0.66-0.82) |
| **Total number of respondents**^3^ | 192 710 | 172 969 |

Abbreviations: OR= Odds ratio; CI= Confidence Interval.

^1^Analyses were performed using DHS sample weights, total number of respondents are presented unweighted. ^2^The age group 50-54 years only includes male participants. ^3^The total number of respondents (n=192,712) differs from the total study sample (n=172,572), as more people answered the “ever tested for HIV” question than the HIVST question.

***Supplemental Digital Content 8 – HIVST use among those who are aware of HIVST (Table)***

**Table 10**

*Multivariable logistic regression analysis with survey fixed effects to examine the association between use of HIVST and participant characteristics among participants who are aware of HIVST^1^*

|  | **Use of HIVST** | |
| --- | --- | --- |
|  | **Model 1** | **Model 2** |
|  | **OR** **(95% CI)** | **OR** (**95% CI)** |
| **Sex** |  |  |
| Men | REF | REF |
| Women | 1.53 (1.35-1.75) | 1.69 (1.47-1.93) |
| **Age** **groups** |  |  |
| 15-19 years | REF | REF |
| 20-24 years | 1.32 (1.08-1.61) | 1.24 (1.01-1.53) |
| 25-29 years | 1.29 (1.05-1.57) | 1.21 (0.98-1.51) |
| 30-34 years | 1.35 (1.08-1.68) | 1.24 (0.97-1.58) |
| 35-39 years | 1.29 (1.03-1.62) | 1.22 (0.96-1.55) |
| 40-44 years | 1.27 (1.00-1.62) | 1.24 (0.96-1.61) |
| 45-49 years | 1.11 (0.85-1.44) | 1.02 (0.76-1.35) |
| 50-54 years^2^ | 1.13 (0.74-1.72 | 1.09 (0.69-1.70) |
| **Residence type** |  |  |
| Urban | REF | REF |
| Rural | 0.86 (0.73-1.03) | 0.88 (0.73-1.06) |
| **Highest educational level** |  |  |
| No education | REF | REF |
| Primary | 0.75 (0.61-0.92) | 0.76 (0.62-0.94) |
| Secondary | 0.98 (0.81-1.18) | 0.99 (0.81-1.20) |
| Higher | 1.38 (1.11-1.71) | 1.25 (1.00-1.56) |
| **Household** **wealth** **index** |  |  |
| Poorest | REF | REF |
| Poorer | 1.06 (0.85-1.32) | 0.98 (0.78-1.24) |
| Middle | 0.86 (0.67-1.09) | 0.81 (0.63-1.04) |
| Richer | 0.89 (0.71-1.13) | 0.86 (0.68-1.10) |
| Richest | 0.76 (0.59-0.98) | 0.78 (0.61-1.01) |
| **Marital status** |  |  |
| Never in union | REF | REF |
| Married | 1.11 (0.96-1.29) | 1.12 (0.96-1.31) |
| Living with partner | 1.13 (0.89-1.44) | 1.08 (0.83-1.39) |
| Widowed | 0.64 (0.39-1.06) | 0.55 (0.32-0.95) |
| Divorced/separated | 1.02 (0.80-1.30) | 0.97 (0.74-1.25) |
| **HIV stigma severity score** |  |  |
| 0 | .. | REF |
| 1 |  | 1.00 (0.75-1.33) |
| 2 |  | 1.09 (0.82-1.44) |
| 3 |  | 1.12 (0.86-1.46) |
| 4 |  | 1.62 (1.20-2.17) |
| 5 |  | 0.49 (0.33-0.71) |
| 6 |  | 0.23 (0.14-0.36) |
| **Total number of respondents** | 23 257 | 20 616 |

Abbreviations: OR= Odds ratio; CI= Confidence Interval.

^1^Analyses were performed using DHS sample weights, total number of respondents are presented unweighted. ^2^The age group 50-54 years only includes male participants.

**Use of HIVST among those who are aware**

Regression analysis showed that women (OR 1.53, 95% CI 1.35-1.75) and higher educated people (no education vs. higher: OR 1.38, 95% CI 1.11-1.71) had greater odds of having ever used HIVST compared to men and people with lower education. Different to the results of HIVST use among the whole population, we found no significant differences between wealth and HIVST use among people who were aware of HIVST. Moreover, people who were perceived to have higher HIV-related stigma, were less likely to have used HIVST among those aware (0 vs. 6: OR 0.23, 95% CI 0.14-0.36), compared to people with lower stigma scores (Supplemental Digital Content 8, Table 10).

***Supplemental Digital Content 9 – HIV prevalence per country (Table)***

**Table 11**

*Overview: HIV prevalence per country*

| **Country** | **HIV prevalence (UNAIDS estimates)**^1^ | **Population size 2017**^2^ | **Calculated HIV prevalence in counts** |
| --- | --- | --- | --- |
| **Burundi** | 1.10% | 10.8 million | 118 800 |
| **Cameroon** | 3.70% | 24.6 million | 910 200 |
| **Guinea** | 1.50% | 12.1 million | 181 500 |
| **Malawi** | 9.60% | 17.7 million | 1 699 200 |
| **Senegal** | 0.40% | 15.4 million | 61 600 |
| **Sierra Leone** | 1.40% | 7.5 million | 105 000 |
| **South Africa** | 18.8% | 57.0 million | 10 716 000 |
| **Zambia** | 11.5% | 16.9 million | 1 943 500 |
| **Zimbabwe** | 13.3% | 14.2 million | 1 888 600 |
| **Sub-Saharan Africa** | 4.12% | 1 050 million | 43 260 000 |

The countries included in this study represent about (17 624 400 / 43 260 000 x 100% =) 40.7% of people living with HIV in the SSA region.

***Supplemental Digital Content 10 – Access to HIVST (Table)***

**Table 12**

*Overview: access to HIVST per country*

| **Country** | **Survey year** | **HIVST accessibility that year** |
| --- | --- | --- |
| **Burundi** | 2016/2017 | 2017: has a supportive policy, but HIVST not yet implemented^1^ |
| **Cameroon** | 2018 | 2018: HIVST policy under development^2^ |
| **Guinea** | 2018 | 2018: HIVST policy under development^2^ |
| **Malawi** | 2015/2016 | 2016: HIVST policy under development^3^ |
| **Senegal** | 2017 | 2018: HIVST policy under development^2^ |
| **Sierra Leone** | 2019 | 2020: HIVST policy under development^4^ |
| **South Africa** | 2016 | 2016: this year HIVST was included as an additional strategy in national HIV testing policy^5^ |
| **Zambia** | 2018 | 2018: HIVST policy implemented^2^ |
| **Zimbabwe** | 2015 | 2016: HIVST policy under development^3^ |

**References**

1 World Health Organization. Burundi - HIV Country Profile 2016 Report. 2017. <https://www.who.int/hiv/data/Country_profile_Burundi.pdf> (accessed March 29, 2021).

2 World Health Organization. HIV self-testing strategic framework: a guide for planning, introducing and scaling-up HIV testing services. 2018.

3 London School of Hygiene & Tropical Medicine. HIV self-testing Africa Initiative - STAR. 2016. https://hivstar.lshtm.ac.uk/2016/04/03/hiv-self-testing-policies/ (accessed March 9, 2021).

4 HIV self-testing research and policy hub. HIV self-testing policy. HIVST.org. 2020. http://hivst.org/ (accessed March 9, 2021).

5 Venter F, Majam M, Jankelowitz L, Adams S, Moorhouse M, Carmona S, et al. South African HIV self-testing policy and guidance considerations. *South Afr J HIV Med* 2017; 18: 775.
